# Three-Chamber Actuated Humanoid Joint-Inspired Soft Gripper: Design, Modeling, and Experimental Validation

**DOI:** 10.3390/s25082363

**Published:** 2025-04-08

**Authors:** Yinlong Zhu, Qin Bao, Hu Zhao, Xu Wang

**Affiliations:** College of Mechanical and Electronic Engineering, Nanjing Forestry University, Nanjing 210037, China; ylzhu@njfu.edu.cn (Y.Z.); qinbao@njfu.edu.cn (Q.B.); zh915760941@163.com (H.Z.)

**Keywords:** soft gripper, humanoid joint actuator, finite element simulation, bending characteristics

## Abstract

To address the limitations of single-chamber soft grippers, such as constant curvature, insufficient motion flexibility, and restricted fingertip movement, this study proposes a soft gripper inspired by the structure of the human hand. The designed soft gripper consists of three fingers, each comprising three soft joints and four phalanges. The air chambers in each joint are independently actuated, enabling flexible grasping by adjusting the joint air pressure. The constraint layer is composed of a composite material with a mass ratio of 5:1:0.75 of PDMS base, PDMS curing agent, and PTFE, which enhances the overall finger stiffness and fingertip load capacity. A nonlinear mathematical model is established to describe the relationship between the joint bending angle and actuation pressure based on the constant curvature assumption. Additionally, the kinematic model of the finger is developed using the D–H parameter method. Finite element simulations using ABAQUS analyze the effects of different joint pressures and phalange lengths on the grasping range, as well as the fingertip force under varying actuation pressures. Bending performance and fingertip force tests were conducted on the soft finger actuator, with the maximum fingertip force reaching 2.21 N. The experimental results show good agreement with theoretical and simulation results. Grasping experiments with variously sized fruits and everyday objects demonstrate that, compared to traditional single-chamber soft grippers, the proposed humanoid joint-inspired soft gripper significantly expands the grasping range and improves grasping force by four times, achieving a maximum grasp weight of 0.92 kg. These findings validate its superior grasping performance and potential for practical applications.

## 1. Introduction

The popularity of soft robots has increased with advancements in new materials and 3D printing technology [1,2,3]. Soft robots possess unlimited degrees of freedom and continuous deformation capabilities, allowing for extensive actions like torsion, bending, and expansion [4,5,6,7]. Soft grippers, a significant branch of soft robotics [8,9], are crafted from flexible materials, in contrast to rigid grippers. They offer clear benefits in handling soft, delicate, and irregular objects [10,11,12,13], and exhibit superior adaptability to various environments, promising exciting applications [14,15,16,17].

In recent years, soft gripper actuators have rapidly developed due to their excellent contact adaptability. H. Du et al. [18] designed a four-finger flexible gripper with an adjustable installation angle based on bending artificial muscles. The gripper’s ability to achieve stable grasping relies on the position and number of its contact points, allowing it to grasp objects weighing up to 1050 g. However, improvements are needed in the grasping range and flexibility of the flexible gripper. Wei et al. [19] developed an adaptive flexible robot gripper featuring variable stiffness and particle interference, which enables the entire soft gripper to alter its stiffness during grasping. Nonetheless, this adaptability comes at the cost of reduced compliance and slower response speed. Zhao et al. [20] proposed a soft gripper with a modular variable stiffness structure. This design embeds flexible hook-and-loop fasteners at the base of the soft actuator and on one side of the variable stiffness chamber, enabling the transition between normal and variable stiffness grasping modes through separation or combination.

Some scholars draw inspiration from the high flexibility, adaptability, and control capabilities of organisms, which has significantly advanced the development of soft actuators that mimic biological characteristics. Inspired by the human hand, Zhang et al. [21] developed TacPalm-SoftHand, which integrates a high-density vision-based tactile palm with dual-segment pneumatic soft fingers. This design enables seamless palm–finger coordination, allowing adaptive grasping of various objects, fine object perception and classification, and precise operations such as card picking, fabric defect detection, and teapot pouring. However, the gripper’s load-bearing capacity still needs improvement. Han Fei et al. [22] proposed a bionic soft actuator featuring a composite chamber with a sealed tube structure, enhancing the response speed of the soft actuator compared to conventional single-channel chamber pressure supplies. Xie et al. [23], inspired by the dexterous tentacles of octopuses, designed a sucker-type bionic octopus soft gripper. However, the actuators of these bionic animals exhibit a single grasping mode and limited flexibility. In contrast, the humanoid hand soft actuator integrates the flexibility and functionality of human hands, enabling it to perform a wide range of grasping tasks as well as fine manipulations [24]. Li et al. [25] developed an all-soft anthropomorphic hand (Dash), which comprises five soft pneumatic fingers and a soft pneumatic palm, allowing for the easy and stable grasping of daily objects of varying shapes and weights. Nevertheless, the stiffness of DASH is not adjustable, leading to a relatively low output force. The thumb of the humanoid hand designed by Hashemi et al. [26] is fixed to a rigid palm without any degree of freedom and can only move along a predetermined trajectory, which significantly restricts the hand’s dexterity. Furthermore, during grasping, the rigid palm fails to effectively conform to the object, thereby diminishing its grasping stability. Sun et al. [27] proposed a three-joint soft actuator with a rigid–flexible coupled humanoid finger structure. Compared to traditional fully soft fingers, it significantly enhances fingertip output force, reaching up to 1.51 N. However, it has not undergone practical grasping tests and remains insufficiently explored in application research.

Currently, humanoid finger actuators typically employ a single-chamber continuous bending structure, which constrains their grasping range, flexibility, and stiffness. To address these limitations, this study presents a soft gripper featuring three-chamber actuation, comprising three soft finger actuators. Each finger is equipped with three joints, each independently actuated by distinct air chambers. The constraint layer is constructed from a composite material of PDMS and PTFE to enhance both stiffness and load-bearing capacity, achieving a maximum fingertip force of 2.21 N. Compared to the design proposed by Sun et al., this represents a 46.3% improvement. Based on the constant curvature assumption, a nonlinear mathematical model is developed to describe the relationship between the bending angle of the soft finger joint and the actuation pressure. The Denavit–Hartenberg (D–H) parameter method is employed to perform a kinematic analysis of the entire finger, further exploring its workspace. Finite element simulations utilizing ABAQUS investigate the effects of varying joint pressures and phalange lengths on the bending deformation and grasping performance of the soft fingers, as well as evaluating the fingertip force under different actuation pressures. Finally, performance tests of the actuator and comparative grasping experiments with traditional single-chamber soft grippers validate the superior bending performance and grasping capability of the proposed design.

## 2. Design and Fabrication

### 2.1. Structural Design

**Figure 1:**
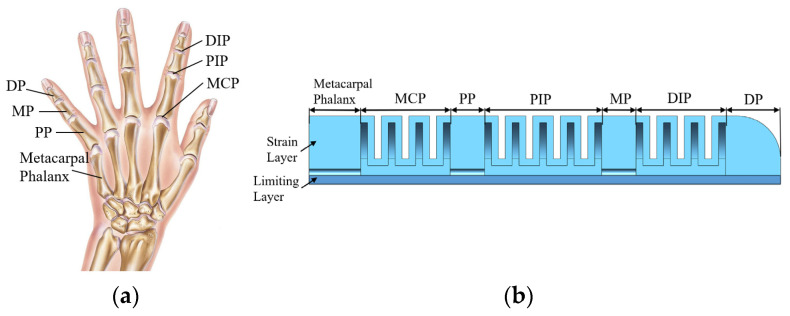
Structural design and manufacturing process of soft finger: (**a**) human finger structure; (**b**) internal structure of soft finger.

The hand, instrumental as a sensory organ in allowing humans to perceive and engage with their surroundings, is celebrated for its flexibility and complexity [28,29]. Figure 1a illustrates the structure of the human hand, with each finger primarily composed of phalanges and joints. To mimic the size parameters of the human middle finger, a humanoid soft finger was designed, as depicted in Figure 1b. This soft finger consists of three curved joints: distal interphalangeal (DIP) joint, proximal interphalangeal (PIP) joint, metacarpophalangeal (MCP) joint, and four phalanges: distal phalanx (DP), middle phalanx (MP), proximal phalanx (PP), and metacarpal phalanx [30]. The joints and phalanges are devised using flexible silicone materials. The joint section segment highlights a pneumatic mesh framework, hosting separate chambers that interlink the three joints.

### 2.2. Fabrication

**Figure 2:**
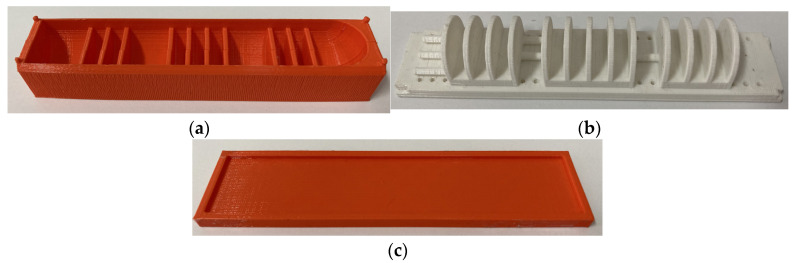
The humanoid soft finger actuator mold: (**a**) the lower mold of the strain layer; (**b**) the upper mold of the strain layer; and (**c**) the mold for the constraint layer.

This study utilizes a MakerBot Print 3D printer to directly print the designed molds, with a printing precision of 0.1 mm. The molds are made of PLA material. As shown in Figure 2a, the lower mold of the strain layer is used to form the actuator’s chamber structure. Due to the relatively long length of the humanoid soft finger actuator, direct demolding during fabrication is challenging; thus, the lower mold is divided into two parts. Figure 2b shows the upper mold of the strain layer, which primarily forms the internal channels of the chamber and the gaps for placing the air tubes. Figure 2c illustrates the mold for the constraint layer of the humanoid finger actuator.

**Figure 3:**
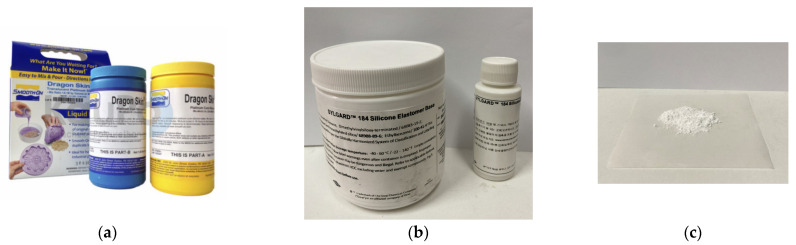
The materials for making the humanoid hand actuator: (**a**) Dragon Skin 20; (**b**) the PDMS base and curing agent; and (**c**) PTFE powder.

The strain layer of the actuator is made of Dragon Skin 20 silicone rubber (Smooth-On, Inc., Macungie, PA, USA), as shown in Figure 3a. A precision electronic scale is used to measure components A and B in a 1:1 mass ratio, followed by thorough mixing. The constraint layer of the actuator is composed of a PDMS/PTFE composite, as shown in Figure 3b,c. PDMS is prepared by mixing the PDMS base and curing agent at a 5:1 mass ratio, while the mass ratio of PDMS to PTFE is determined in Section 4.2.1.

**Figure 4:**
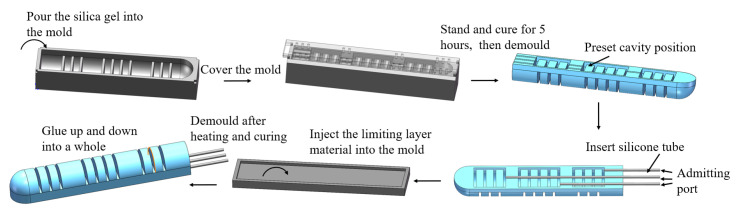
Manufacturing process of soft finger.

The detailed fabrication process of the humanoid soft finger actuator is illustrated in Figure 4. First, the thoroughly mixed Dragon Skin 20 is slowly poured into the lower mold shown in Figure 2a. Once the chamber is completely filled, the upper mold in Figure 2b is carefully placed on top, and the silicone material is left to cure at room temperature for 5 h. After curing, the strain layer is demolded, and three pre-prepared silicone air tubes are embedded into the designated chamber positions. The contact surfaces between the air tubes and the strain layer are firmly bonded with silicone. Next, the uniformly mixed constraint layer material is slowly poured into the mold shown in Figure 2c and leveled with a tool to ensure uniform thickness. After curing for 5 h, the constraint layer is demolded. Finally, the strain layer and the constraint layer are bonded together with silicone to form a tight integration, completing the fabrication of the humanoid soft finger.

**Figure 5:**
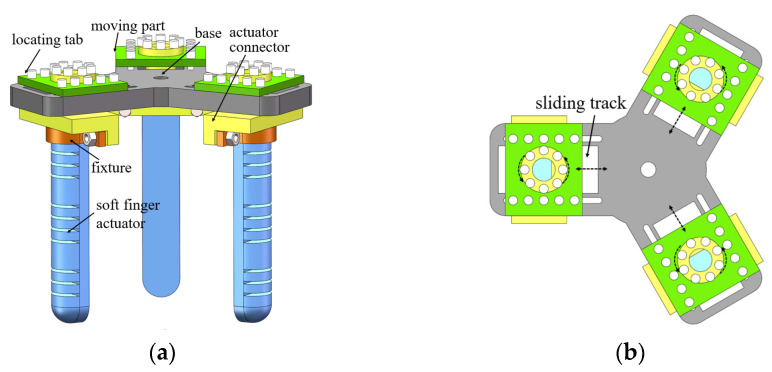
Whole structure of soft gripper: (**a**) structural design of soft gripper; (**b**) top view of soft gripper.

The soft gripper is equipped with three humanoid soft fingers to effectively handle objects. It comprises soft fingers, fixtures, connectors, moving parts, and bases, as depicted in Figure 5. The fixture and base provide a secure grip while the adjustable connecting and moving elements effectively cater for an array of object forms and dimensions. The soft fingers are crafted from soft materials, while all other components are constructed from PLA materials, quickly manufactured using a 3D printer. Refer to Table 1 for detailed structural parameters of the soft finger.

## 3. Mathematical Modeling and Kinematics Analysis

Due to differences in structural stiffness, only the joints bend during the inflation process, while the four phalanges of the soft fingers exhibit minimal bending. To facilitate analysis, it is assumed that each joint maintains a constant curvature, and the four phalanges are treated as rigid bodies. This allows for the establishment of a mathematical relationship between the bending angle of the soft finger joints and air pressure. Subsequently, the rigid motion of the four phalanges and the bending motion of the three joints can be comprehensively analyzed to establish a kinematic model for each finger of the humanoid joint-inspired soft gripper.

### 3.1. Mathematical Model of Joint Bending

**Figure 6:**
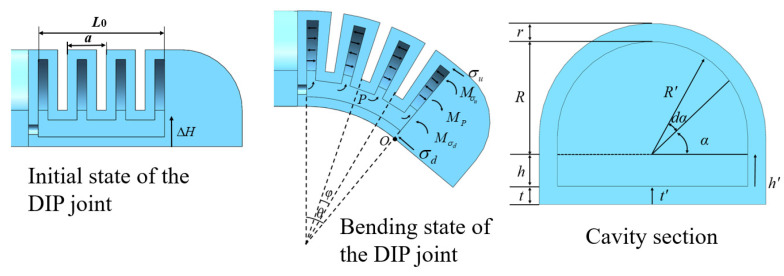
Schematic diagram of soft actuator before and after deformation.

The curvature of the chambers within each joint of the soft finger was maintained as constant, culminating in the formation of a curved arc segment by connecting short arc segments of each chamber pair. The MCP joint and the DIP joint consist of four pairs of chambers, while the PIP joint consists of five pairs. This paper utilizes a piecewise constant curvature model to analyze the joints based on their deformation characteristics. For instance, considering the DIP joint of the actuator, the internal air pressure is denoted as *P*, the bending angle of a pair of chambers as *φ*, the bending central angle of the actuator in its deformed state as *θ*, and the overall bending angle of the joint as *θ*/2. The bending model established is illustrated in Figure 6, with *R*, *h*, *r*, and *t* representing the cross-sectional radius, height, wall thickness, and actuator limiting layer thickness of the semi-circular chamber, respectively, along with the relevant geometric relationships:

(1) $$L=L_{0}+\Delta L,\;\theta =\frac{L_{0}}{a}\varphi ,\;\Delta L=\theta \Delta H$$

where *L*_0_ represents the initial length of the joint part of the humanoid finger actuator in an undeformed state; *L* is the axial length of the deformed joint part of the humanoid finger actuator; Δ*L* indicates the length change of the joint part of the humanoid finger actuator before and after deformation; Δ*H* is the offset distance of the horizontal section of the humanoid finger actuator relative to the bottom surface; and ‘*a*’ corresponds to the length value of a pair of chambers.

Silicone rubber is considered an incompressible material, requiring a strain energy density function to describe its mechanical behavior. The Yeoh model [31] was utilized in this study to analyze the strain layer of a humanoid finger actuator, with its simplified typical binomial parameter:

(2) $$W=C_{10}(I_{1}-3)+C_{20}{\left(I_{1}-3\right)}^{2}$$

The strain energy density function W is defined as a function of strain energy, where *I*_1_ = *λ*_1_^2^ + *λ*_2_ ^2^ + *λ*_3_^2^, with *λ*_1_, *λ*_2_, and *λ*_3_ representing the principal draw ratios in the axial, radial, and thickness directions, respectively. Parameters *C*_10_ and *C*_20_ are associated with the Yeoh model for silicone rubber.

In the context of significant deformation in the strain layer of a soft finger, the Yeoh model proves to be a suitable choice for modeling. The principal stress σiu (i = 1,2,3) can be determined by calculating the partial derivative of the strain energy function with respect to the principal elongation ratio:

(3) $$\sigma_{iu}=\lambda_{iu}\frac{\partial W}{\partial \lambda_{iu}}-p=2{\lambda_{iu}}^{2}\cdot \left[C_{10}+2C_{20}\left(I_{1}-3\right)\right]-p$$

The tensile ratio of the strained layer in all directions is represented by *λ_iu_*, while the stress in all directions of the strain layer is denoted by *σ_iu_*. When considering the bending deformation of a soft finger joint, it is assumed that the strain layer remains undistorted in the radial direction, leading to a radial tension ratio of *λ*_2u_ equal to 1 and a corresponding radial stress of *σ_2u_* equal to 0. The hydrostatic pressure, denoted as *p*, can be determined under incompressible conditions. Therefore, the hydrostatic pressure *p* can be calculated using Formula (3) as follows:

(4) $$p=2\lambda_{2u}^{2}\cdot \left[C_{10}+2C_{20}\left(I_{1}-3\right)\right]$$

When the actuator is bent and deformed, the thickness of the silica gel layer will decrease. Assuming that the axial elongation ratio *λ*_1u_ is *λ*_u_, the radial and circumferential elongation ratio of the corresponding strain layer is ${\lambda_{2u}}^{2}={\lambda_{3u}}^{2}=1/\lambda_{u}$. At the same time, considering that the circumferential stress *σ*_3u_ is much smaller than the axial stress *σ*_1u_, it can be concluded that the axial stress *σ*_1u_ is the only principal stress *σ*_u_ in the strain layer.

The relationship between the axial stress and the axial elongation ratio of the strained layer is obtained from the Formulas (3) and (4):

(5) $$\sigma_{u}=2\left({\lambda_{u}}^{2}-1/\lambda_{u}\right)\cdot \left[C_{10}+2C_{20}\left({\lambda_{u}}^{2}+2/\lambda_{u}-3\right)\right]$$

(6) $$\lambda_{u}=L/L_{0}=(L_{0}+\theta h^{\prime })/L_{0}$$

The deformation of the actuator’s limiting layer is minimal. For the sake of simplification in analysis, it is assumed that the radial strain of the limiting layer is disregarded, and the Neo-Hookean model is employed to characterize the mechanical behavior of the limiting layer. The strain energy density function *W* is defined as follows:

(7) $$W=\frac{\mu }{2}(I_{1}-3)$$

where *μ* is the initial shear modulus of the material.

Since the radial strain of the confining layer is assumed to be neglected, the radial stretching ratio *λ*_2d_ is equal to 1. Due to the small tensile range of the limiting layer, the radial stress *σ*_2d_ is much lower than *σ*_1d_. As a result, *σ*_1d_ is regarded as the primary stress in the length direction of the confining layer and is represented as *σ*_d_.

The length and width dimensions of the limiting layer material are significantly greater than its thickness dimension, suggesting that there is negligible stress in the thickness direction of the actuator’s limiting layer (i.e., *σ*_3d_ = 0). The relationship between axial stress and axial strain of the actuator’s limiting layer, based on the Neo-Hookean model, can be derived using Formula (7):

(8) $$\sigma_{d}=\lambda_{d}\frac{\partial W}{\partial \lambda_{d}}-p=\mu ({\lambda_{d}}^{2}-\frac{1}{\lambda_{d}^{2}})$$

(9) $$\lambda_{d}=L/L_{0}=(L_{0}+\theta t^{\prime })/L_{0}$$

The axial stress *σ*_u_ and *σ*_d_ represent the calculated stresses on the effective sections of the strain layer and the limiting layer of the soft finger actuator. In Figure 4, *M*_P_ is the driving moment of pressure *P* acting on the end boundary of the chamber, *M*_u_ is the resistance moment generated by the partial stress *σ*_u_ in the strained layer, and *M*_d_ is the resistance moment generated by the partial stress *σ*_d_ in the confined layer. The equilibrium state of the chamber joint can be expressed as [32]: *M*_P_ = *M*_u_ + *M*_d_

Here, the driving moment and the resistance moment are defined as follows:

(10) $$M_{p}=\int_{0}^{h}2PR(h^{\prime }+t)dh^{\prime }+2\int_{0}^{\frac{\pi }{2}}\int_{0}^{R}P(R^{\prime }\sin\alpha +h+t)R^{\prime }d\alpha dR^{\prime }$$

(11) $$M_{u}=\int_{0}^{h}2\sigma_{u}R(h^{\prime }+t)dh^{\prime }+2\int_{0}^{\frac{\pi }{2}}\int_{R}^{R+r}\sigma_{u}(R^{\prime }\sin\alpha +h+t)R^{\prime }d\alpha dR^{\prime }$$

(12) $$M_{d}=\int_{0}^{t}2\sigma_{d}(R+r)t^{\prime }dt^{\prime }$$

In the above formula, *h’* is the distance between the cross section of the strain layer of the chamber and the upper surface of the confinement layer, *R’* is the distance from the center of the chamber to the edge of the chamber, and *t’* is the distance between the cross section of the confinement layer of the chamber and the O point.

From Equations (10)–(12), the relationship between the actuation pressure *P* and the strain layer stress *σ*_u_, as well as the constraint layer stress *σ*_d_, can be obtained. Furthermore, from Equations (5), (6), (8) and (9), the relationship between *σ*_u_, *σ*_d_, and the bending angle *θ*/2 can be determined. Therefore, the relationship between the actuation pressure *P* and the bending angle *θ*/2 of the soft finger joint can be derived as follows:

(13) $$P=\frac{\sigma_{u}\left[4+3\pi (h+t)\right]\left[{\left(R+r\right)}^{3}-R^{3}\right]+6\sigma_{u}(2t+h)rh+6\sigma_{d}(R+r)t^{2}}{4R^{3}+3\pi (h+t)R^{2}+6R(h^{2}+2ht)}$$

**Figure 7:**
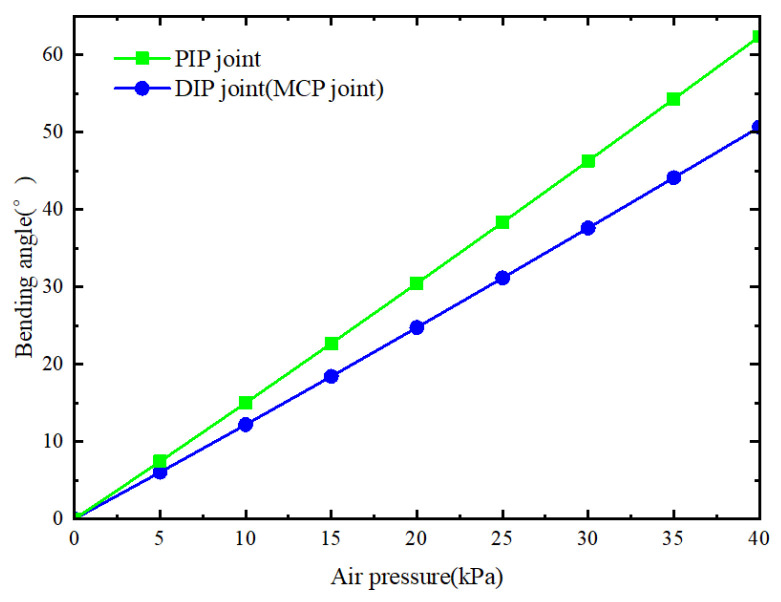
Theoretical bending angle of three joints under different air pressures.

Theoretical bending angles of three bending joints were calculated by substituting the parameters in Table 1 into Formula (11) and setting the air pressure range to 0–40 kPa. The results are depicted in Figure 7. Since the length and other parameters of the MCP joint and the DIP joint are equal, their bending angles are also equal. However, the PIP joint is longer than the other two joints, resulting in a larger bending angle.

### 3.2. Kinematics Analysis of Soft Finger

#### 3.2.1. Kinematics Analysis

**Figure 8:**
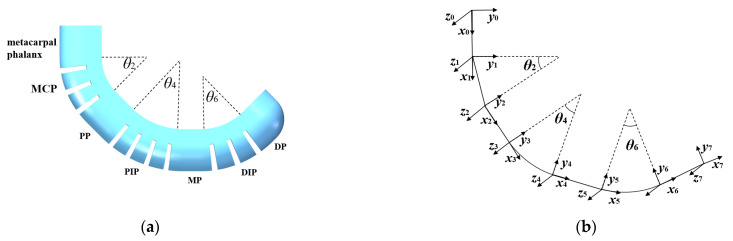
Kinematics diagram of soft finger actuator: (**a**) bending model of actuator; (**b**) establishment of coordinate system.

A kinematics analysis of a soft finger is conducted based on joint bending modeling and phalanx rigid body motion. The kinematics model of the soft finger is established using the standard D–H method, with the coordinate system shown in Figure 8. In this model, only the joints of the soft finger bend with bending angles *θ*_i_/2 (*i* = 2,4,6), while the phalanges remain unbent with bending angle *θ_i_* = 0 (*i* = 1,3,5,7). The soft finger is capable of bending in one direction and moving in a plane, resulting in a torsion angle *α_i_* of 0 for both phalanges and joints.

The lengths of the metacarpal phalanx, proximal phalanx, middle phalanx, and distal phalanx are denoted as *l*_1_, *l*_3_, *l*_5_, and *l*_7_ respectively. The bending angles are represented by *θ*_1_, *θ*_3_, *θ*_5_, and *θ*_7_, all of which are set to 0. Each phalanx rotates by an angle *θ_i_* (where *i* = 1,3,5,7) along the *z*_i−1_ axis, and translates by a distance of *l_i_* along the *x_i_* axis to establish the coordinate system transformation matrix of the phalanx.

(14) Tii−1=cosθi−sinθi⋅cosαisinθi⋅sinαilicosθisinθicosθicosαi−cosθisinαilisinθi0sinαicosαidi0001

By bringing the parameters into simplification, the coordinate system transformation matrix of the phalanx is obtained as follows:

(15) Tii−1=cosθi−sinθi0licosθisinθicosθi0lisinθi00100001

**Figure 9:**
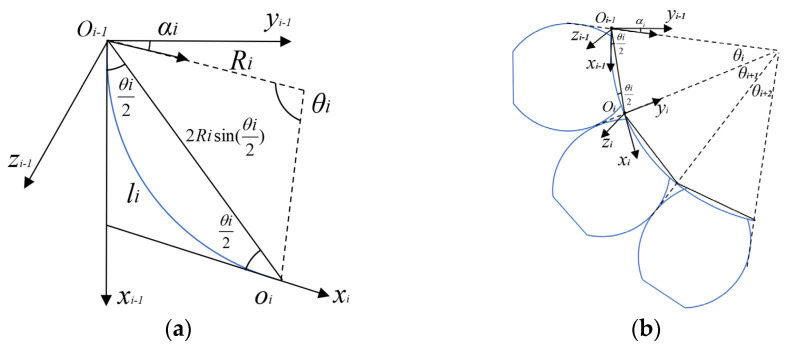
Schematic diagram of motion modeling of joint part of actuator: (**a**) section of arc element bending plan; and (**b**) establishment of joint segment coordinate system.

The establishment of the joint part’s coordinate system is based on the center line of the actuator limiting layer, as illustrated in Figure 9. The soft joint can undergo a transformation from the coordinate system *o_i_*_−1_-*x_i_*_−1_*y_i_*_−1_z*_i_*_−1_ to the coordinate system *o_i_*-*x_i_y_i_*z*_i_* through the following steps: ① Rotate *α_i_* around the *x_i_*_−1_ axis; ② Rotate *θ_i_*/2 around the new *z_i_*_−1_ axis; ③ Translate *o_i_*_−1_*o*_*𝑖*_ forward on the new *x_i_*_−1_ axis; ④ Rotate *θ_i_*/2 around the new *z_i_*_−1_ axis; ⑤ Rotate *α_i_* around the new *x_i_*_−1_ axis.

The radius of curvature of the MCP joint, PIP joint, and DIP joint are *R*_2_, *R*_4_, and *R*_6_ respectively. The bending angles are *θ*_2_/2, *θ*_4_/2, and *θ*_6_/2, respectively (all can be obtained from Section 3.1). The chord length of the arc corresponding to the joint bending angle *θi*/2 (*i* = 2,4,6) is ‖*o_i_*_−1_*o*_𝑖_‖ = 2*R_i_*sin(*θ_i_*/2). Using the D–H parameter method, the relative position and direction between joint coordinate systems are described. The homogeneous coordinate transformation matrix of the bending joint is then obtained.

(16) Tii−1=Rx(αi)Rz(θi2)Tx(li)Rz(θi2)Rx(−αi)=cosθi−cosαisinθi−sinαisinθiRisinθicosαisinθisin2αi+cos2αicosθisin2αi(cosθi−1)22Ricosαisin2(θi2)sinαisinθisin2αi(cosθi−1)2cos2αi+sin2αicosθi2Risinαisin2(θi2)0001

The torsion angle *α_i_* around the *x* axis is 0, and it is simplified by substituting into Formula (14) to obtain the homogeneous coordinate transformation matrix of the bending joint:

(17) Tii−1=Rx(0)Rz(θi2)Tx(li)Rz(θi2)Rx(0)=cosθi−sinθi0Risinθisinθicosθi02Risin2(θi2)00100001

The coordinate transformation matrices of the metacarpal phalanx, metacarpophalangeal joint, proximal phalanx, proximal interphalangeal joint, middle phalanx, distal interphalangeal joint, and distal phalanx are multiplied sequentially and connected to determine the final position of the soft finger actuator.

(18) T=T10T21T32T43T54T65T76=a11a120a14a21a220a2400100001

in which

$$\begin{array}{l} a_{11}=-\cos\theta_{6}(\sin\theta_{2}\sin\theta_{4}-\cos\theta_{2}\cos\theta_{4})-\sin\theta_{6}(\cos\theta_{2}\sin\theta_{4}+\cos\theta_{4}\sin\theta_{2});\\ a_{12}=\sin\theta_{6}(\sin\theta_{2}\sin\theta_{4}-\cos\theta_{2}\cos\theta_{4})-\cos\theta_{3}(\cos\theta_{2}\sin\theta_{4}+\cos\theta_{4}\sin\theta_{2});\\ a_{14}=10\cos\theta_{2}-10\sin\theta_{2}\sin\theta_{4}-15\cos\theta_{6}(\sin\theta_{2}\sin\theta_{4}-\cos\theta_{2}\cos\theta_{4})+R_{2}\sin\theta_{2}-15\sin\theta_{6}(\cos\theta_{2}\sin\theta_{2}+\cos\theta_{4}\sin\theta_{2})\\ +10\cos\theta_{2}\cos\theta_{4}-2R_{4}\sin^{2}\frac{\theta_{4}}{2}\sin\theta_{2}+R_{4}\cos\theta_{2}\sin\theta_{4}-2R_{6}\sin^{2}\frac{\theta_{6}}{2}(\cos\theta_{2}\sin\theta_{4}+\cos\theta_{4}\sin\theta_{2})\\ -R_{6}\sin\theta_{6}(\sin\theta_{2}\sin\theta_{4}-\cos\theta_{2}\cos\theta_{4})+15;\\ a_{21}=\cos\theta_{6}(\cos\theta_{2}\sin\theta_{4}+\cos\theta_{4}\sin\theta_{2})-\sin\theta_{6}(\sin\theta_{2}\sin\theta_{4}-\cos\theta_{2}\cos\theta_{4});\\ a_{22}=-\cos\theta_{6}(\sin\theta_{2}\sin\theta_{4}-\cos\theta_{2}\cos\theta_{4})-\sin\theta_{6}(\cos\theta_{2}\sin\theta_{4}+\cos\theta_{4}\sin\theta_{2});\\ a_{24}=10\sin\theta_{2}+2R_{2}\sin^{2}\frac{\theta_{2}}{2}+15\cos\theta_{6}(\cos\theta_{2}\sin\theta_{4}+\cos\theta_{4}\sin\theta_{2})-15\sin\theta_{6}(\sin\theta_{2}\sin\theta_{4}-\cos\theta_{2}\cos\theta_{4})\\ +10\cos\theta_{2}\sin\theta_{4}+10\cos\theta_{4}\sin\theta_{2}+2R_{4}\sin^{2}\frac{\theta_{4}}{2}\cos\theta_{2}+R_{4}\sin\theta_{2}\sin\theta_{4}-2R_{6}\sin^{2}\frac{\theta_{6}}{2}(\sin\theta_{2}\sin\theta_{4}-\cos\theta_{2}\cos\theta_{4})\\ +R_{6}\sin\theta_{6}(\cos\theta_{2}\sin\theta_{4}+\cos\theta_{4}\sin\theta_{2}). \end{array}$$

#### 3.2.2. Working Space Analysis

**Figure 10:**
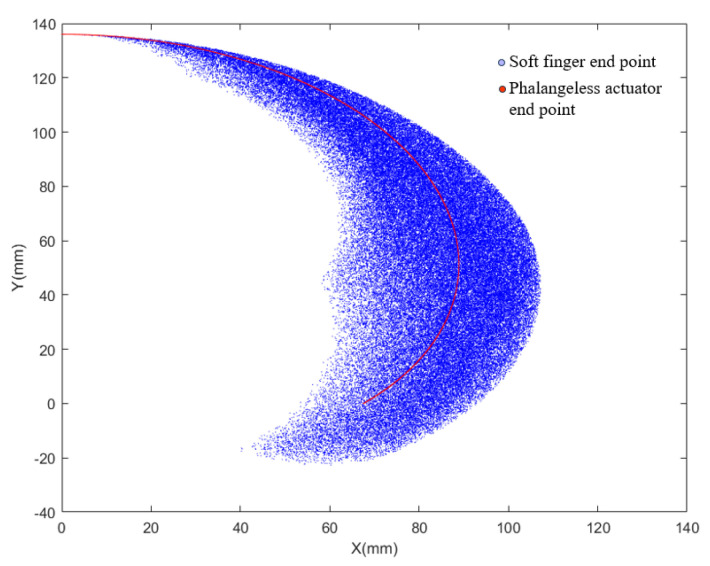
Track of the endpoint of the actuator.

Through ABAQUS simulation, it was determined that the bending central angles of the three joints in the soft finger are approximately 90°, 110°, and 90° under an air pressure of 40 kPa. To meet the bending requirements of each joint in the human hand, the working ranges for the three joints of the soft finger were set at (0,90°), (0,110°), and (0,90°), respectively. Additionally, a continuous phalangeless actuator was introduced for comparison, with a working range set at (0,180°). By programming to address the issue, the results are illustrated in Figure 10. The x-axis exemplifies the displacement of the endpoint during the bending motion of the actuator, and the y-axis symbolizes the direction from the actuator’s base towards the fingertip, effectively representing all motion paths followed by the endpoint of the actuator.

The figure illustrates the trajectories of the endpoints of two types of actuators: the soft finger actuator in blue and the continuous phalangeless actuator in red. The endpoint of the continuous phalangeless actuator follows an arc trajectory, while the endpoint of the soft finger actuator forms a plane area comprising multiple arcs. This visual comparison highlights that the working range of the soft finger actuator is significantly broader than that of the continuous actuator, providing distinct advantages in grasping objects.

## 4. Simulation Analysis and Performance Test

### 4.1. Simulation Analysis

**Figure 11:**
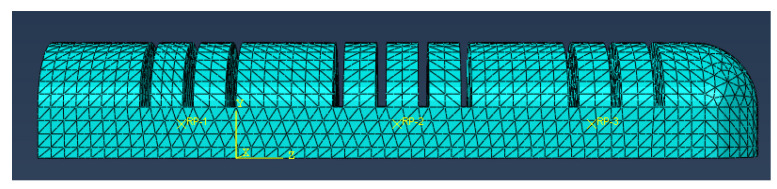
Grid diagram of soft finger actuator.

**Figure 12:**
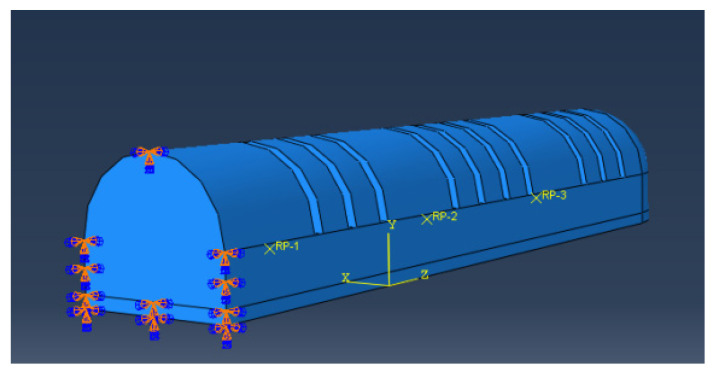
Constraints on the inflatable end.

(1) The soft finger actuator was designed using SolidWorks 2018 and subsequently imported into ABAQUS to create the finite element simulation analysis model. In the grid attribute module, to accommodate the complex structure of the actuator and enhance the convergence of the simulation model, all actuators were simplified into a quadratic hybrid entity unit (C3D10H), as illustrated in Figure 11.

(2) The entire soft finger actuator is classified as a superelastomer. The strain layer of the soft finger was composed of Dragon Skin 20 silica gel, with material coefficients C10 and C20 under the Yeoh model being 0.11 and 0.02, respectively. Specific tensile test data can be referenced in [33]. The limiting layer was constructed from PDMS/PTFE with a mass ratio of 8:1, and the material parameter under the Neo-Hookean model was 0.962 [33].
(3) Constraints were established at the location of the inflation port of the actuator to ensure that it remained unaffected during the bending deformation process. The fingertip served as the free end, allowing for an accurate representation of the inflation deformation process of the humanoid finger driver, as illustrated in Figure 12.

(4) The properties of the fluid chamber were set and the pressure of the fluid chamber on the inner walls of the three bending joints of the actuator was established. During the process of applying air pressure to the chamber of the soft finger actuator, the outer wall of the joint section of the chamber expands and deforms, causing the adjacent chamber walls to come into contact and compress against each other. Therefore, it was essential to implement self-contact on the surfaces that may interact to prevent the chamber walls from penetrating one another, which could lead to non-convergence of the simulation. Additionally, the mechanical properties of the self-contact were configured to exhibit frictionless and normal hard contact behavior.

#### 4.1.1. Simulation Analysis of Phalanx Length

The length of the finger’s phalanx impacts the deformation profile of the actuator when bending, subsequently affecting the grasping performance of the soft finger. To investigate this influence, phalanx lengths of *l* = 5 mm, *l* = 10 mm, and *l* = 15 mm were compared with a continuous phalangeless model (*l* = 0).

**Figure 13:**
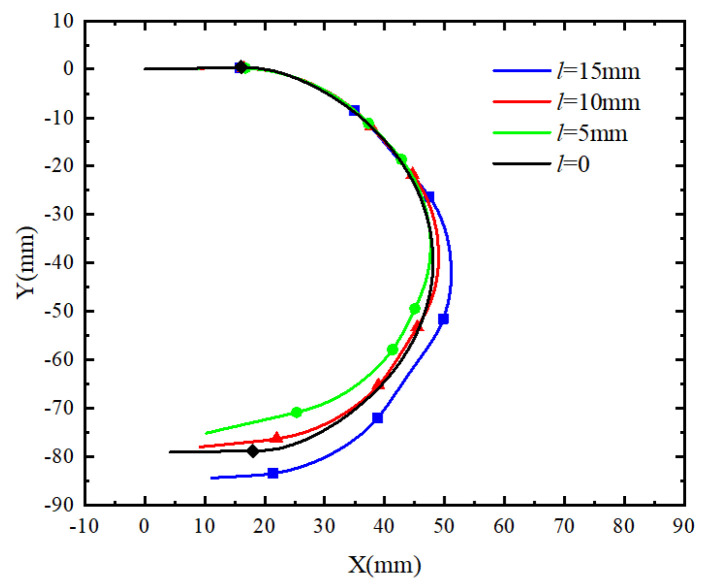
Bending profile of actuators with different phalanx lengths.

Four models were simulated and analyzed under pressure ranging from 0 to 40 kPa. The deformation curve of the actuator at 30 kPa was extracted, and the bending deformation contour effect diagram of the four models was obtained (see Figure 13). Among them, the actuator with a phalanx length of 5 mm exhibited the smallest bending deformation profile. As the phalanx length increased, the bending deformation profile also increased, indicating a stronger bending deformation ability of soft fingers. Additionally, the bending deformation profile of the continuous phalangeless actuator was larger than that of the 5 mm actuator and equivalent to that of the 10 mm actuator. Considering that longer phalanx parts result in a greater total length and weight of the actuator, which may not be beneficial for improving the grasping ability of the soft gripper, the phalanx length of the soft finger was determined to be 10 mm. 

**Figure 14:**
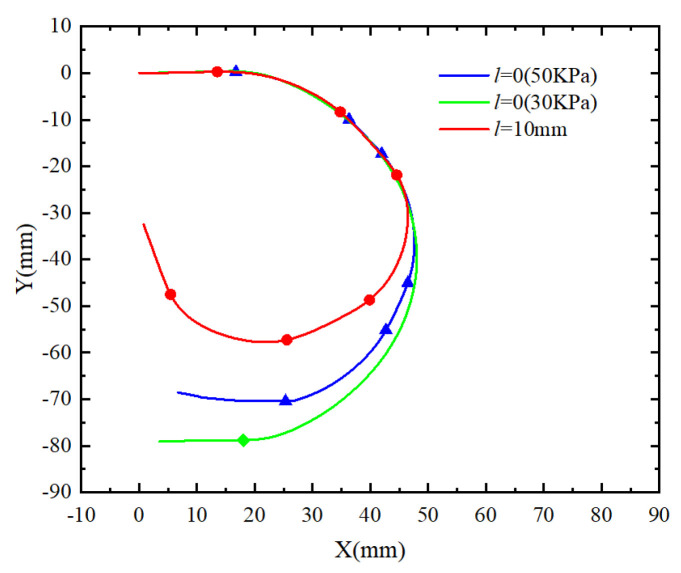
Simulation bending profile of two actuators under different air pressures.

In order to further compare the bending deformation ability of the soft finger actuator with a phalanx length of 10 mm and a phalangeless actuator under varying air pressures, additional simulation analysis was conducted. The air pressure at the DIP joint of the soft finger actuator was set to 50 kPa, while the PIP joint and MCP joint were set to 40 kPa and 30 kPa, respectively. For the phalangeless actuator, air pressures of 30 kPa and 50 kPa were applied sequentially. The X and Y coordinates of the nodes in the restricted layer were extracted to obtain the simulated bending profiles of the two actuators under different air pressures, as illustrated in Figure 14.

When exposed to varying air pressures, the flexible finger actuator demonstrates a larger bending angle and greater bending deformation capability compared to the phalangeless actuator. In contrast to the phalangeless soft actuator, the soft finger actuator features three independent bending joints. By adjusting the air pressure in these joints, the contact surface during object manipulation is increased, enabling stable and flexible grasping of objects with diverse shapes. This enhanced grasping ability offers a competitive advantage.

#### 4.1.2. Simulation Analysis of Soft Finger Bending

**Figure 15:**
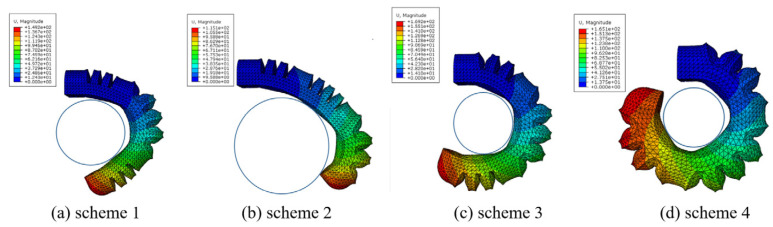
Simulation bending displacement of different joints with air inflation.

The study focuses on analyzing the diameter of a ball that can be grasped by soft fingers under different joint pressures using ABAQUS simulation. Air pressure is applied to the bending joint of the actuator according to the scheme in Table 2. The soft finger is then simulated using the finite element method, resulting in a bending displacement diagram as shown in Figure 15.

In schemes 1 and 2, the soft finger envelope grasping diameter is φ32 mm and φ45 mm, respectively. The joint’s inflation pressure is low, and the bending curvature radius is large. However, at low air pressure, the actuator’s output force decreases, leading to a lower load capacity and inability to grasp heavy objects. Therefore, the low air pressure scheme is more suitable for grabbing large and lightweight items. In schemes 3 and 4, the envelope grasping diameter is φ28 mm and φ24 mm, respectively. The three bending joints exhibit strong inflation pressure, high load capacity, and a small bending radius, making them ideal for grasping small yet heavy items. Depending on the target objects to be grasped, adjusting the air pressure of the three independent bending joints of the soft finger allows for flexible grasping and enhances the gripping capability of the soft gripper.

#### 4.1.3. Simulation Analysis of Terminal Force

**Figure 16:**
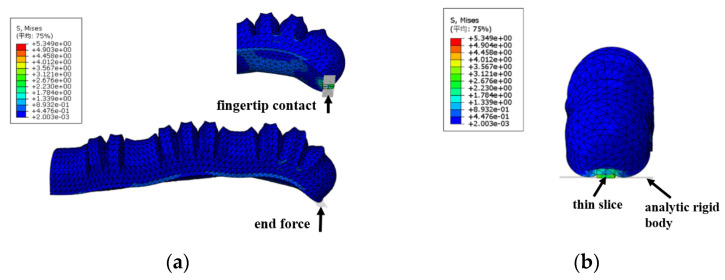
Simulation of actuator contact: (**a**) overall contact diagram; (**b**) terminal contact state diagram.

The impact of the force applied by the soft finger on an object is crucial in assessing the actuator’s grasping ability. To maximize the end force output during bending, an analytical rigid body (positioned at 0°) is introduced beneath the fingertip of the actuator’s distal phalanx. Contact properties between this analytical rigid body and the fingertip are then configured for force contact analysis. Additionally, a sheet with a high elastic modulus is placed under the soft finger’s fingertip and attached to the actuator’s end. This setup allows the sheet to come into surface-to-surface contact with the analytical rigid surface, resulting in the generation of end force, as illustrated in Figure 16.

**Figure 17:**
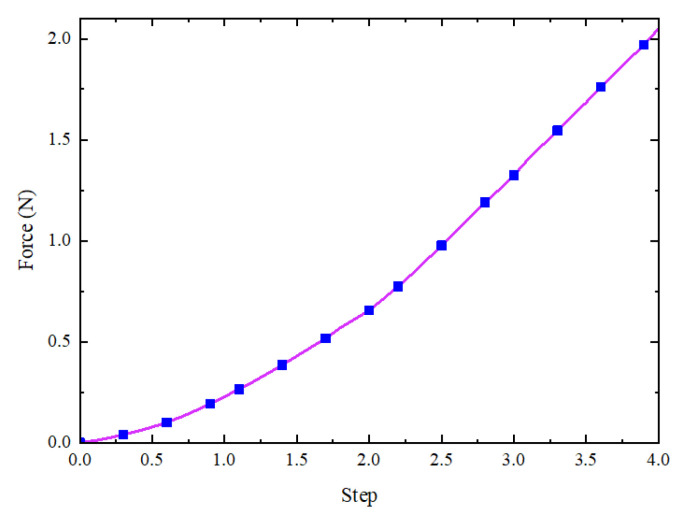
Simulation of end force on soft finger.

The CFN (Total Force Due to Contact Pressure) of the contact pair between the thin slice and the analytical rigid body is determined from the History Output in the post-processing module. When the pressure in the three joint chambers is uniform and the simulated pressure is set between 0 and 40 kPa, the maximum output end force of the actuator can reach 2.05 N, as illustrated in Figure 17.

The advantage of a multi-joint soft finger lies in its capacity to independently regulate the pressure of each chamber, thereby effectively enhancing the grasping force at the fingertip when manipulating complex objects. A simulation analysis was performed by varying the air pressures in the three independent chambers, with the resulting terminal forces summarized in Table 3. Schemes 1 to 3 investigate the influence of adjusting the air pressure of the distal joint while maintaining the other joints at 20 kPa. Similarly, schemes 1, 4, and 5 focus on the air pressure of the proximal joint, while schemes 1, 6, and 7 examine the modifications in the air pressure of the metacarpal joint, all under the condition of keeping the other joints at 20 kPa. The data presented in Table 3 clearly demonstrate that increasing the air pressure in a single joint can enhance the fingertip force; however, the extent of improvement varies based on which joint is pressurized. Notably, alterations in the air pressure of the distal joint exert the most significant influence on the terminal force. When the air pressures of the proximal, middle, and distal joints are set to 20 kPa, 20 kPa, and 40 kPa, respectively, the terminal force attains a value of 1.402 N.

### 4.2. Performance Test of Humanoid Joint-Inspired Soft Gripper

#### 4.2.1. Uniaxial Tensile Test of PDMS/PTFE Composites

**Figure 18:**
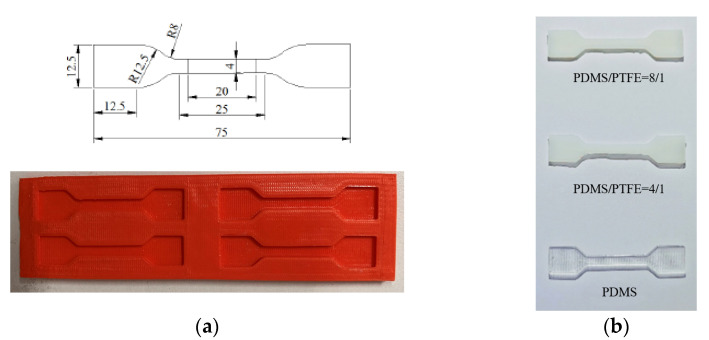
Tensile sample diagram: (**a**) tensile test sample mold; (**b**) dumbbell-shaped samples of different ratios.

This paper proposes PDMS and PTFE as the constraint layer materials for the humanoid soft finger actuator. By adjusting the mass ratio of these two materials, the stiffness of the actuator can be modified, thereby affecting its bending angle. To investigate the effect of different ratios on the mechanical properties of the material, a uniaxial tensile test was conducted. Three different sample compositions were prepared: pure PDMS (PDMS base/curing agent = 5:1), PDMS/PTFE = 4:1 (i.e., PDMS base/curing agent/PTFE = 5:1:1.5), and PDMS/PTFE = 8:1 (i.e., PDMS base/curing agent/PTFE = 5:1:0.75). During the sample preparation process, PDMS base, curing agent, and PTFE were mixed according to the predetermined mass ratios (total mass approximately 15 g). The mixture was stirred for 10 min, poured into a 3D-printed sample mold, and left undisturbed for 10 min to eliminate air bubbles. The mold was then placed in a vacuum drying oven at 40 °C for 3 h for curing. To minimize the influence of environmental factors, samples were prepared at different time intervals, and defective samples were discarded. Five dumbbell-shaped tensile specimens were fabricated for each composition, as shown in Figure 18.

**Figure 19:**
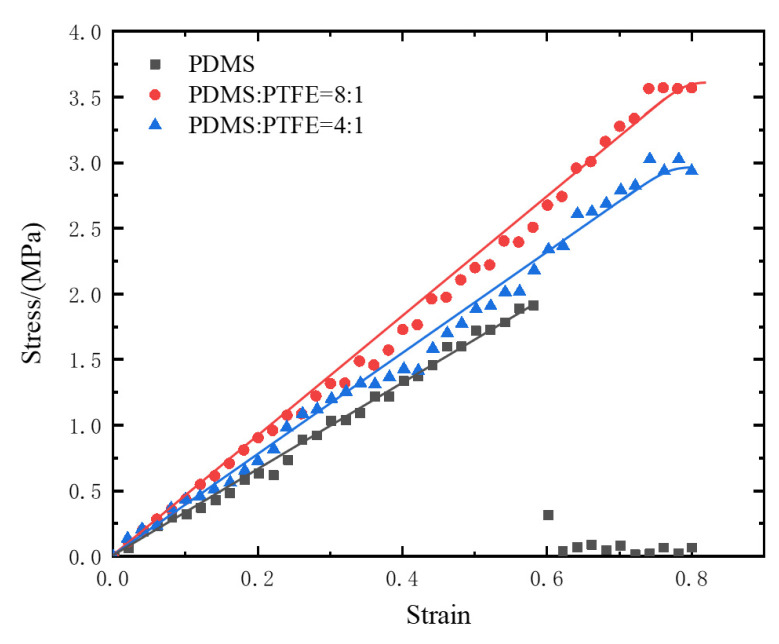
Stress and strain tensile test results.

The stress–strain curves obtained from the uniaxial tensile tests are shown in Figure 19. When the strain exceeded 0.58, the stress of the pure PDMS sample dropped sharply due to fracture. Under the same strain conditions, the sample with a lower PTFE content (mass ratio 8:1) exhibited higher stress than the sample with a higher PTFE content (mass ratio 4:1), indicating a higher material modulus and better resistance to fracture. This meets the requirements for the constraint layer material. Therefore, this study selected the PDM/SPTFE composite material with a mass ratio of 8:1 (i.e., PDMS/curing agent/PTFE = 5:1:0.75) as the constraint layer for the humanoid soft finger actuator.

#### 4.2.2. Flexural Performance Test of Soft Finger

**Figure 20:**
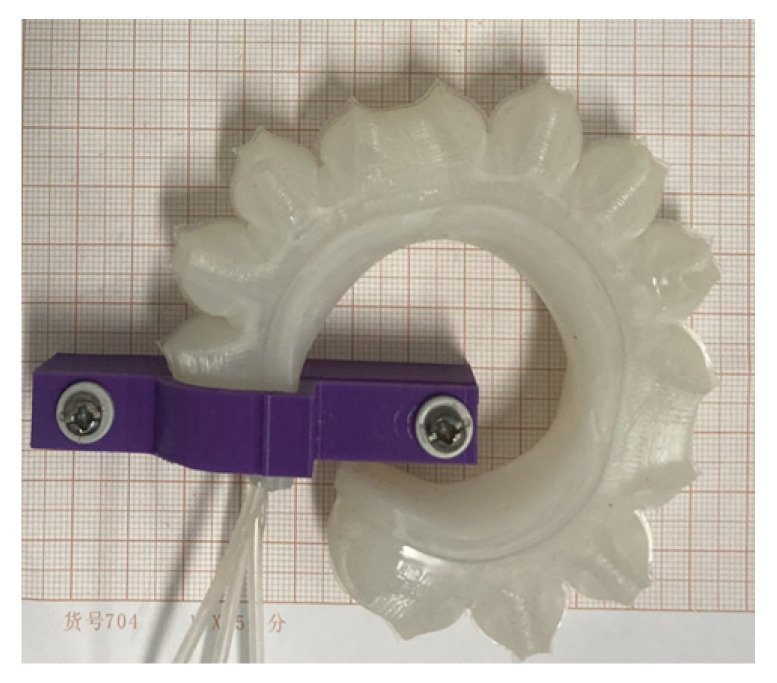
Deformation of soft finger.

The soft finger is carefully positioned on the coordinate paper while simultaneously adjusting the air pressure of the proportional valves for the three joints within the range of 0–40 kPa. The resulting deformed soft finger can be observed in Figure 20.

**Figure 21:**
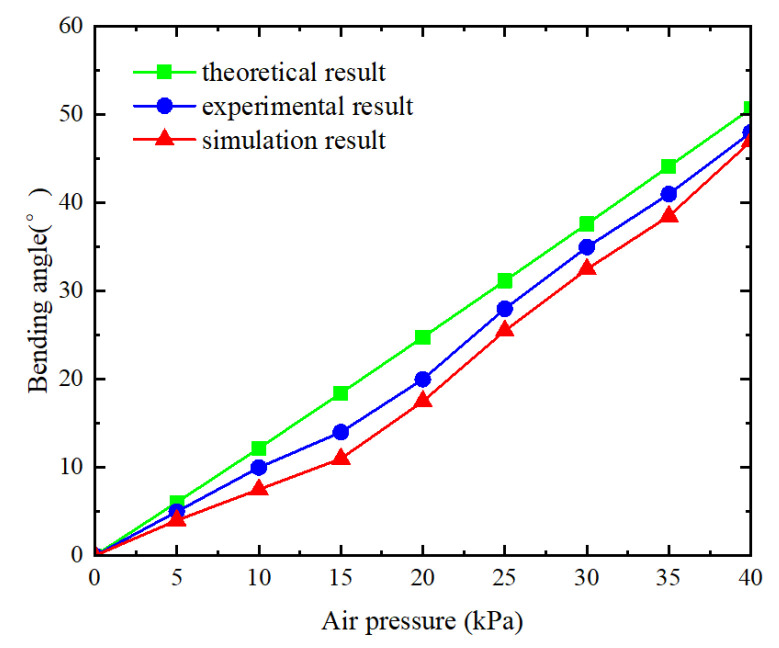
The relationship between the bending angle and the air pressure.

The bending angle of the DIP joint was measured at 5 kPa intervals and compared to both theoretical and simulation results, as illustrated in Figure 21. For air pressures ranging from 0 to 20 kPa, the bending angle gradually increases with minimal deformation. Beyond 20 kPa, the bending angle rises rapidly along with increasing deformation. The synergy between internal pressure and contact with the outer wall significantly amplifies the actuator’s bending performance under elevated pressure conditions. Overall, the experimental, theoretical, and finite element analysis results exhibit similar trends and are in close agreement. However, discrepancies in the results can be attributed to certain assumptions made during theoretical modeling.

**Figure 22:**
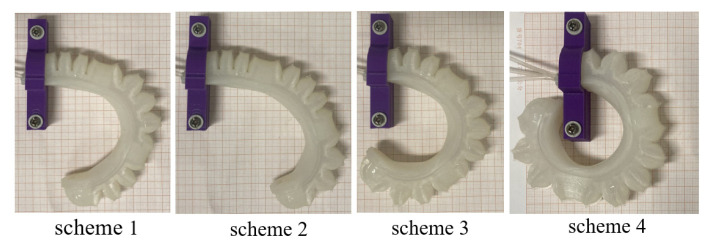
Bending displacement experiment of different joints with air inflation.

In order to compare with the simulation results in Figure 15, the experiment applied air pressure to three joints based on the scheme outlined in Table 2. The resulting deformation diagram of the soft finger is depicted in Figure 22. Inspection of the figure substantiates that the bending deformation of the soft finger corresponds closely with the simulation results.

**Figure 23:**
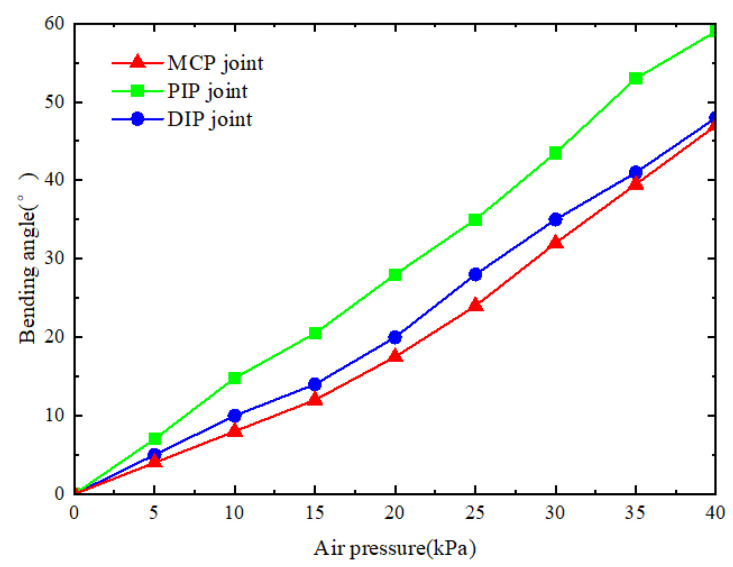
The relationship between the bending angle of the three joints and the air pressure.

The bending angles of each joint of the soft finger were recorded every 5 kPa, ranging from 0 to 90°. The results, as shown in Figure 23, indicate that the bending angles of the MCP joint and DIP joint follow the same trend due to the equal number of chambers. In contrast, the PIP joint, with more chambers, exhibits stronger bending ability under the same air pressure, reaching a bending capacity of 59° at 40 kPa. The deformation trend aligns closely with the theoretical bending angle in Figure 5, confirming the model’s accuracy.

#### 4.2.3. Terminal Force Test of Soft Finger

**Figure 24:**
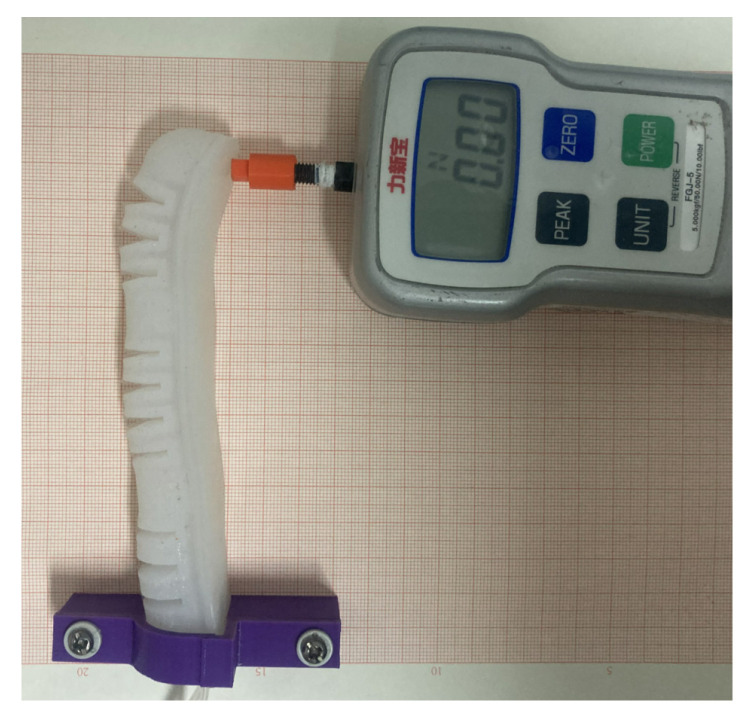
Terminal force test of actuator.

To evaluate the load capacity of the soft finger actuator and the accuracy of the simulation results, the terminal force of the soft finger actuator was tested. The test device is illustrated in Figure 24. In the initial uninflated state, the dynamometer was securely positioned at the point where the bending angle of the actuator is zero, ensuring that the contact point of the dynamometer is perpendicular to the end of the soft finger actuator. Concurrently, the inflation pressure of the three joints of the actuator was adjusted, and the terminal force of the soft finger actuator was recorded as the air pressure varied from 0 to 40 kPa.

**Figure 25:**
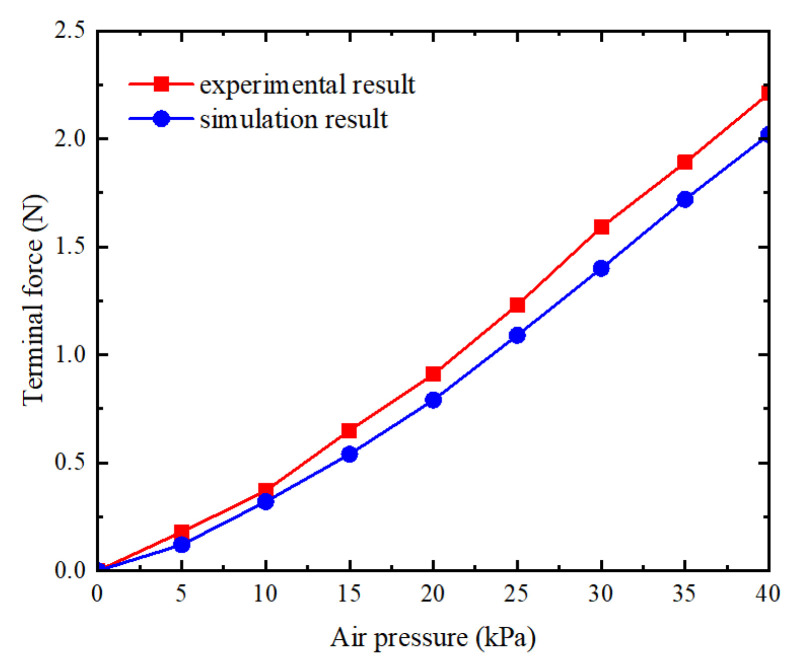
Comparison of experimental and simulation result of terminal force.

As shown in Figure 25, a comparison of the experimental results with the simulation results reveals that the simulation of terminal force aligns with the overall trend observed in the experimental data. During the joint bending process of the soft finger actuator, the terminal force increases as the air pressure rises. Furthermore, the maximum terminal force obtained from the test is 2.21 N, and the maximum deviation from the simulation results is about 7%.

#### 4.2.4. Trajectory Test of Endpoint of Soft Finger

**Figure 26:**
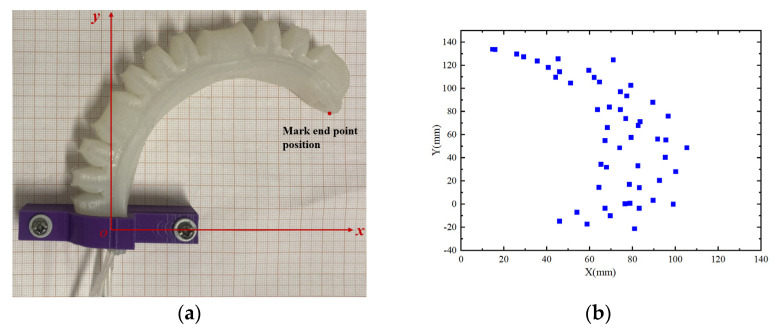
Motion trajectory test of actuator endpoint: (**a**) marked endpoint position; (**b**) test result.

The numerical solution for the motion space of the endpoint of the soft finger actuator was obtained in Section 3.2. This section aims to test the actuator to verify the accuracy of the theoretical results. As illustrated in Figure 26a, the experimental setup secures the soft finger actuator onto a coordinate paper, establishing the *xoy* coordinate system. In this system, the x-axis represents the offset distance of the endpoint during the bending movement of the actuator, while the y-axis indicates the direction from the base of the actuator towards the fingertip. Concurrently, three joints are inflated, with the air pressure set within a range of 0 to 40 kPa. To maximize data collection, the position coordinates of the endpoint are recorded at intervals of 2 kPa. All collected data are then plotted as a scatter plot, yielding the test results presented in Figure 26b. The trajectory of the endpoint of the soft finger actuator, as shown in the figure, is largely consistent with the theoretical trajectory depicted in Figure 8.

#### 4.2.5. Grasping Performance Test of Soft Gripper

**Figure 27:**
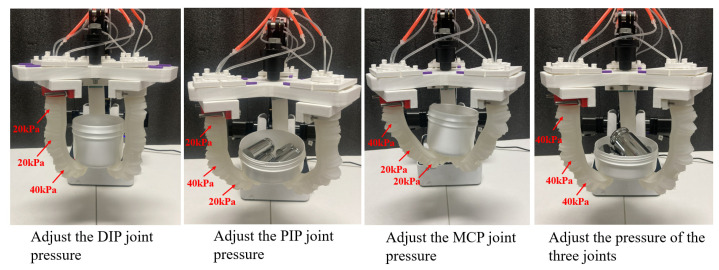
Adjustment of grasping of each joint.

To assess the grasping ability of the soft gripper under varying air pressures, a disk grasping method was employed for testing. Successively, disks with diameters of 60 mm, 80 mm, and 100 mm were utilized, with ample space for weight installation. The test procedure involved increasing the air pressure in each joint chamber of the soft finger, followed by gently placing weights into the disk once stable grasping was achieved, as depicted in Figure 27. This procedure was replicated for every disk size, and the aggregate weight of the objects gripped by the soft gripper under varying joint pressures was documented.

Given that the overall stiffness of soft fingers is relatively low, their grasping performance under a pressure of 15 kPa is suboptimal. In this paper, one joint was selected to adjust the air pressure, and the air pressure of the other joints was kept around 20 kPa. In relation to grasping, the grasping weight of the soft gripper under different air pressures could be obtained by slowly adding weights, as shown in Table 4. From the grasping results, it can be known that adjusting the air pressure of the DIP joint of the soft gripper has the greatest influence on the grasping performance while keeping the inflation pressure of the PIP joint and the MCP joint constant. When the air pressure of three joints is 40 kPa, the soft gripper can grab 0.92 kg objects.

**Figure 28:**
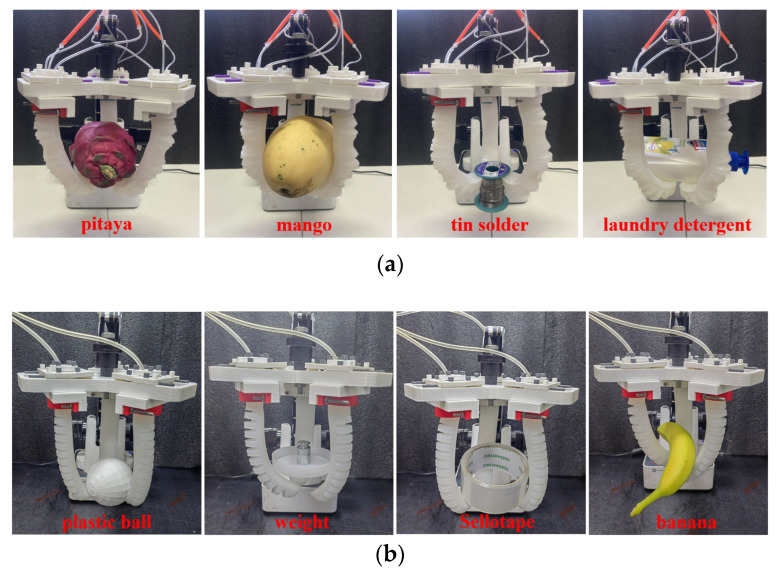
Grasping objects test: (**a**) humanoid joint-inspired soft gripper; (**b**) continuous phalangeless gripper.

Objects of varying shapes and weights were selected for grasping, and a continuous phalangeless gripper was introduced for comparison, as illustrated in Figure 28. The corresponding joint air pressures are presented in Table 5 and Table 6, respectively. Table 7 compares the performance of the humanoid joint-inspired soft gripper and the continuous phalangeless gripper across three dimensions: object shape adaptability, load capacity, and object surface roughness and friction. The results indicate that the humanoid joint-inspired soft gripper outperforms the continuous phalangeless gripper in adapting to different shapes, enhancing grasping stability and increasing load capacity. This advantage is primarily attributed to its multi-joint design, which optimizes force distribution and improves grasping performance.

## 5. Conclusions

1. This paper presents a humanoid joint-inspired soft gripper actuated by a three-chamber system. Each soft finger is independently driven by separate air chambers, with a constraint layer composed of a PDMS base, PDMS curing agent, and PTFE in a mass ratio of 5:1:0.75. Experimental results demonstrate that a single soft finger can generate a maximum fingertip force of 2.21 N, while the three-finger assembly can grasp objects weighing up to 0.92 kg, including mangoes, detergent bottles, and soldering wire. These results underscore the design’s excellent adaptability and stable grasping capability across various shapes and sizes.
2. Based on the constant curvature assumption and moment balance principle, this study employs the Yeoh and Neo-Hookean models to establish a nonlinear mathematical model for the distal joint of the soft finger, with experimental validation confirming its accuracy. Additionally, kinematic analysis is conducted using the D–H parameter method to calculate the fingertip position and orientation. A comparison with a phalangeless actuator reveals that the proposed design offers a larger workspace and greater flexibility.
3. In the ABAQUS simulation analysis, the effects of different pressure combinations on the grasping ability of the soft fingers are explored, along with comparisons for phalange lengths of 5 mm, 10 mm, and 15 mm. Results indicate that the phalangeless model exhibits poor bending capability, while a phalange length of 10 mm provides the best grasping performance and structural stability while maintaining a low weight. Furthermore, within the actuation pressure range of 0–40 kPa, the simulated maximum fingertip force reaches 2.05 N, with a maximum deviation of approximately 7% from the experimental results, thereby verifying the reliability of the proposed design.

In future work, we will continue to optimize the overall structure of the soft finger to enhance the control accuracy of joint angles and the stability of grasping force, thereby achieving more precise and stable grasping operations. Additionally, by integrating sensor technology, we aim to enable multi-modal perception in the soft gripper, improving its ability to detect object shape, hardness, and other characteristics, which will further enhance grasping performance. The application of the soft gripper will be expanded to a broader range of fields, including medical rehabilitation and service robots, exploring its potential in various scenarios.

## Figures and Tables

**Table 1:** Structure parameters of soft finger actuator.

Parameters	Values	Parameters	Values
metacarpal phalanx length *l*_1_/mm	15	DP length *l*_7_/mm	15
MCP length *l*_2_/mm	26	section diameter *R*/mm	10.5
PP length *l*_3_/mm	10	chamber thickness *r*/mm	2
PIP length *l*_4_/mm	34	chamber height *h*/mm	3.5
MP length *l*_5_/mm	10	limiting layer thickness *t*/mm	2
DIP length *l*_6_/mm	26	total weight *G*/g	53

**Table 2:** Pressure application scheme for three joints.

Scheme	MCP/kPa	PIP/kPa	DIP/kPa
1	10	40	10
2	10	10	40
3	20	40	20
4	40	40	40

**Table 3:** End forces of soft finger under different inflation schemes.

Scheme	MCP/kPa	PIP/kPa	DIP/kPa	Maximum End Force/N
1	20	20	20	0.741
2	20	20	30	0.974
3	20	20	40	1.402
4	20	30	20	0.835
5	20	40	20	1.285
6	30	20	20	0.706
7	40	20	20	1.136

**Table 4:** Grasp weight of soft gripper.

MCP Pressure/kPa	PIP Pressure/kPa	DIP Pressure/kPa	Weight/g
10	10	10	20
20	20	20	130
30	30	30	450
40	40	40	920
20	20	25	200
20	20	30	380
20	20	35	480
20	20	40	720
20	25	20	180
20	30	20	280
20	35	20	350
20	40	20	420
25	20	20	150
30	20	20	250
35	20	20	300
40	20	20	360

**Table 5:** Grasping objects and the corresponding joint pressure of the soft gripper.

Scheme	MCP/kPa	PIP/kPa	DIP/kPa	Weight/g
pitaya	35	30	30	391.1
mango	40	40	40	714.9
tin solder	20	20	30	206.7
laundry detergent	30	30	40	496.5

**Table 6:** Grasping objects and the corresponding pressure of the phalangeless gripper.

Scheme	MCP/kPa	Weight/g
plastic ball	13	391.1
weight	20	714.9
Sellotape	17	206.7
banana	25	496.5

**Table 7:** Comparison of humanoid joint-inspired soft gripper and continuous phalangeless gripper.

Comparison Dimension	Humanoid Joint-Inspired Soft Gripper	Continuous Phalangeless Gripper
Object Shape Adaptability	By adjusting air pressure at three joints (MCP, PIP, DIP), it conforms better to object surfaces and adapts to irregularly shaped objects.	The limited grasping range makes it difficult to fully conform to non-spherical or irregularly shaped objects, increasing the risk of slippage.
Load Capacity	The higher stiffness allows for a maximum grasping weight nearly four times that of the single-chamber gripper, enabling stable grasping of heavier objects.	Structural limitations prevent independent joint control, making it difficult to securely grasp heavier objects.
Object Surface Roughness and Friction	The adjustable joint pressure increases the contact area with the object surface, enhancing friction and making it suitable for smooth or slippery objects (e.g., detergent bottles).	Poor adaptability makes it difficult to achieve good contact with low-friction surfaces, leading to lower grasping stability and a higher risk of slippage.

## Data Availability

All test data mentioned in this paper will be made available upon request from the corresponding author’s email with appropriate justification.
